# Supplementary material for: Wind Speed and Landscape Context Mediate Campylobacter Risk among Poultry Reared in Open Environments
Source: Animals (Basel). 2023 Jan 31;13(3):492. doi: 10.3390/ani13030492 (PMC9913591; doi:10.3390/ani13030492)
Supplement: Supplementary file 1 [file animals-13-00492-s001.zip › animals-2053276-supplementary.pdf]

Supplementary Materials

Wind speed and landscape context mediate *Campylobacter* risk among poultry reared in open environments

Olivia M. Smith, Kevin A. Cornell, Michael S. Crossley, Rocio Crespo, Matthew S. Jones, William E. Snyder, and Jeb P. Owen

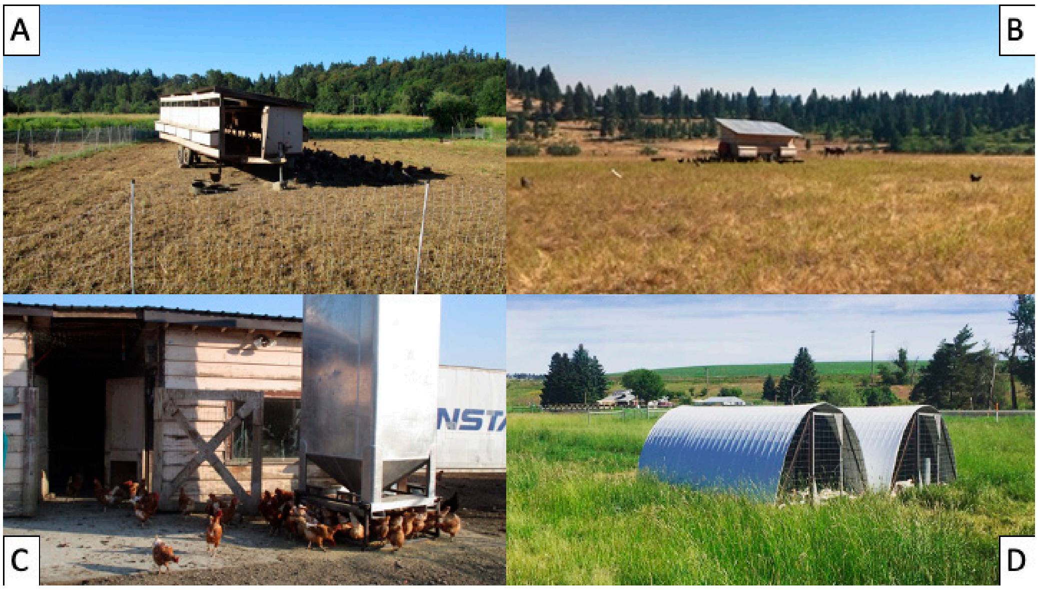

Figure S1. Photos of open-environment poultry rearing practices used by farms included in this study. (**a**) Pasture-raised, rotational layer flock on a non-certified farm that uses organic practices. (**b**) Free ranging layer flock with mobile coop. This flock was fed a non-organic GMO-free diet but did not receive any chemical treatments, antibiotics, or vaccinations. (**c**) A stationary flock from a certified organic farm. (**d**) A broiler flock in an enclosed, mobile pen. This flock was rotated 3 – 4 times a week, received no anticoccidials or antibiotics, and was fed a conventional grain diet.

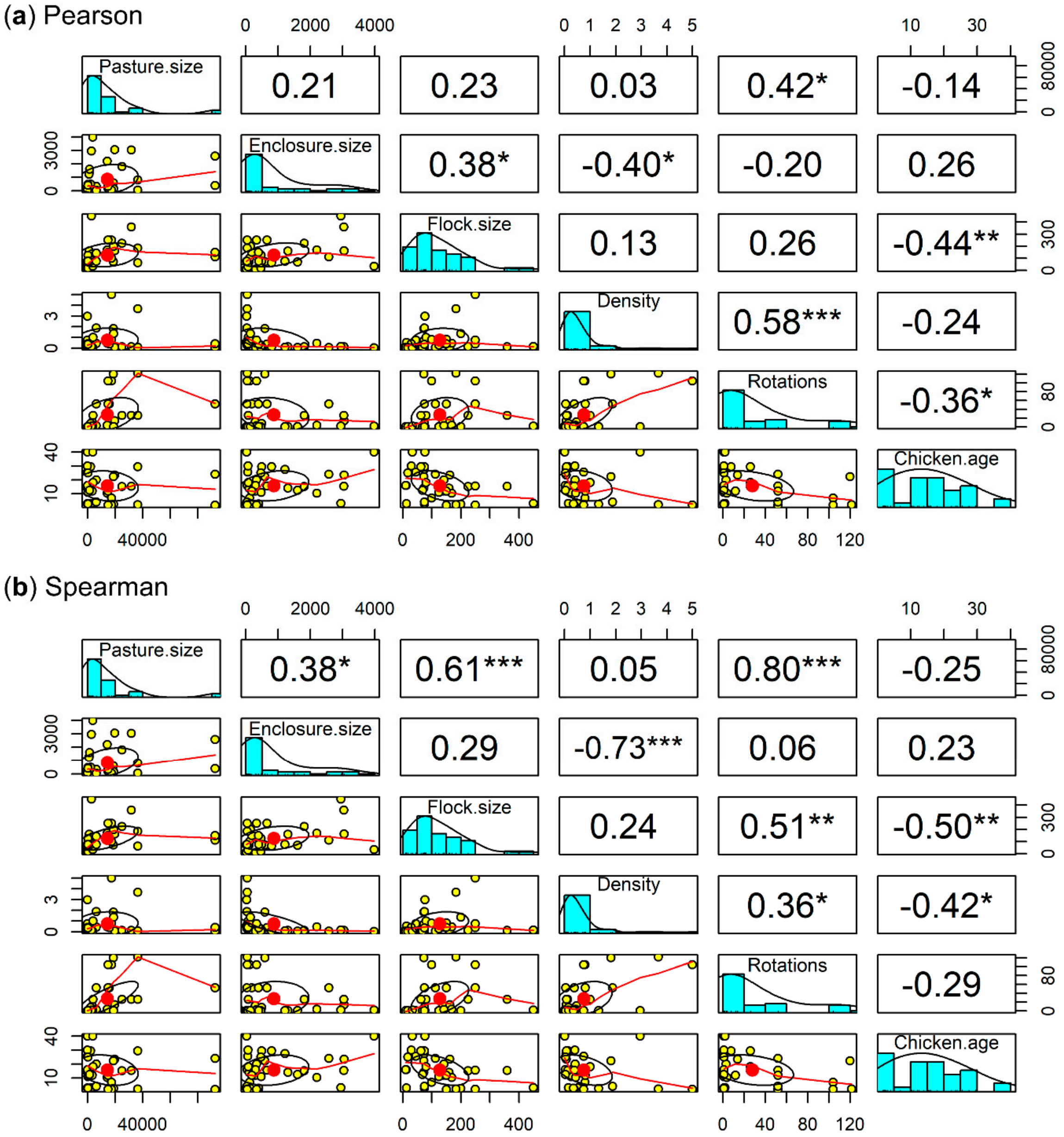

Figure S2. (**a**) Pearson's and (**b**) Spearman's pairwise correlation plots between 6 farm management variables including pasture size (m^2; space ever available to chickens throughout year), enclosure size (m^2; area used by chickens at time of survey), flock size (number of chickens per flock), density (flock size/enclosure size), rotations per year (times moved per year), and average chicken age for the flock (months).

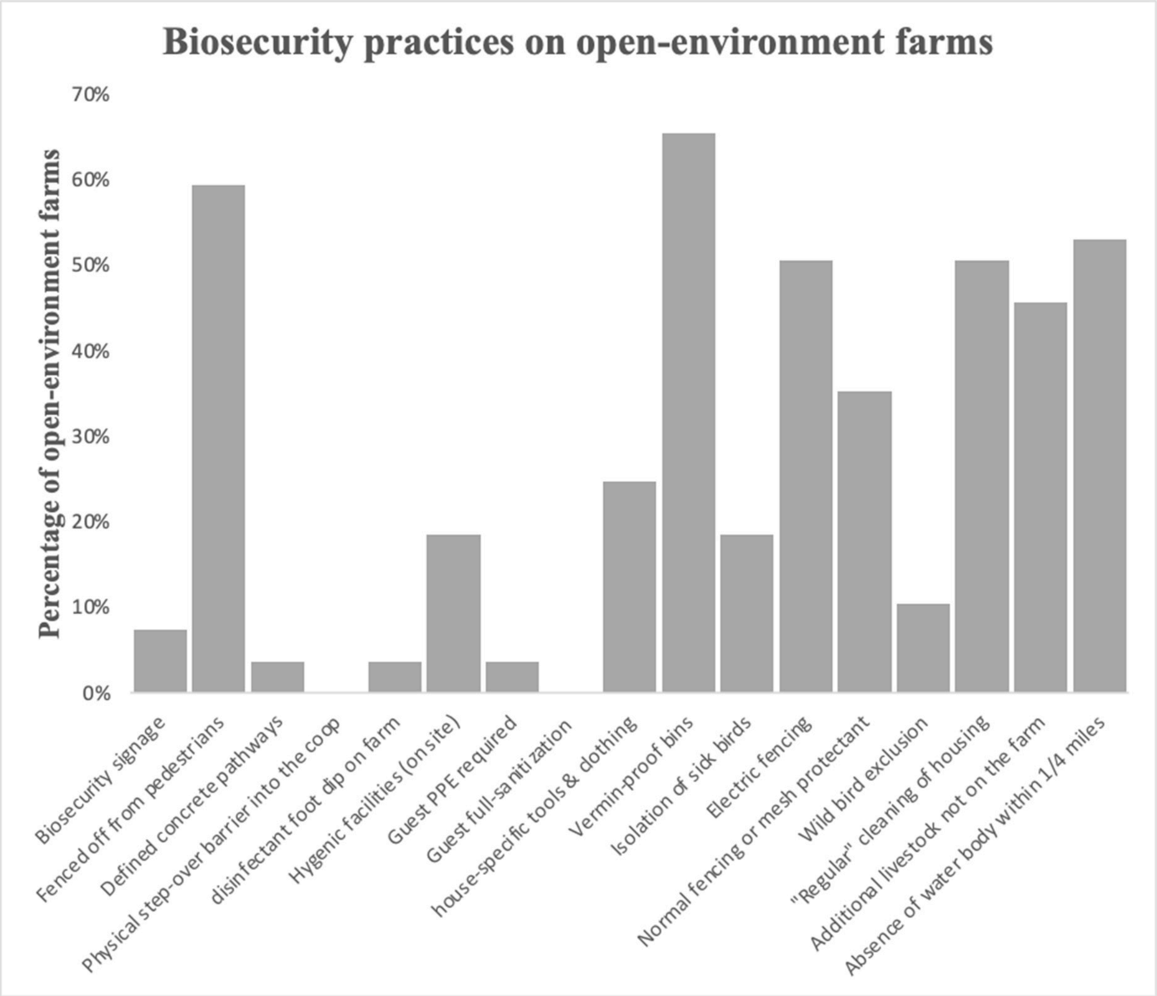

Figure S3. Percentage of farms that used each biosecurity practice documented. We quantified biosecurity of farms using questions relevant to biosecurity practices on open-environment farms. The survey included 17 yes or no questions, giving each farm a biosecurity score of 0 to 17. Generally, the open-environment farms included in this study had poor biosecurity practices (4.5 ± 0.42). Three conventional farms surveyed for biosecurity practices using the same criteria scored much higher (scores: 10, 12, and 14) for comparison.

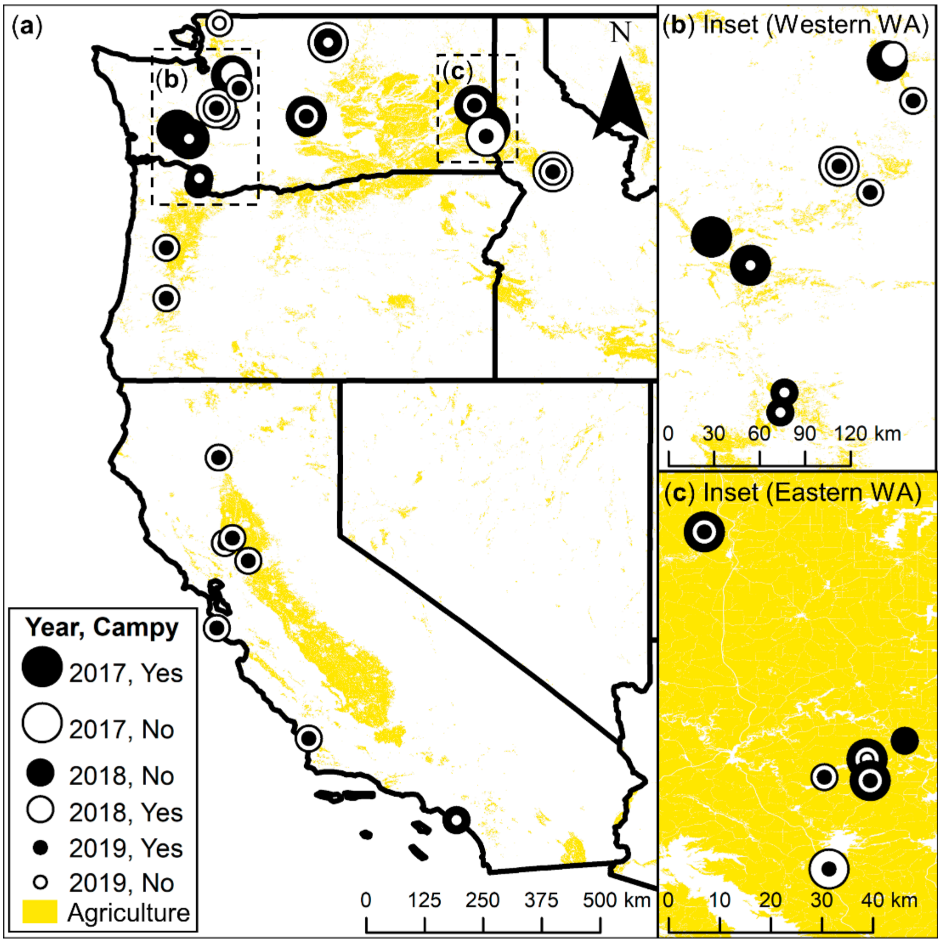

Figure S4. Map showing locations of *Campylobacter* spp. positive (black) and negative (white) farms surveyed for each year of the study (2017–2019) in the USA states of California, Oregon, Washington, and Idaho. (**a**) All farms, (**b**) inset of a Western Washington farm cluster, and (**c**) inset of an Eastern Washington farm cluster. Areas the insets correspond to are indicated on (**a**) by black, dashed boxes. Year(s) each farm was surveyed indicated by circle size: large = 2017; medium = 2018; small = 2019. Yellow background indicates agricultural land use.

Table S1. Full candidate model set including fixed effects, random effects, and notes. Our candidate model set included potential single, additive, and interactive effects of on- and off-farm variables hypothesized to influence *Campylobacter* spp. prevalence in poultry feces, in addition to a null (random effects only) model.

Model	Fixed	Random	Notes
1 (null)		(1|Farm:Flock)	
2	Wild bird contact + Rotational (y/n)	(1|Farm:Flock)	
3	% agriculture	(1|Farm:Flock)	
4	Wind speed	(1|Farm:Flock)	
5	Production type PC1	(1|Farm:Flock)	
6	Production space PC2	(1|Farm:Flock)	
7	Soil organic matter	(1|Farm:Flock)	Excluded from final analyses due to missing data
8	Temperature	(1|Farm:Flock)	
9	Humidity	(1|Farm:Flock)	
10	Production type PC1 * Wild bird contact + Rotational (y/n)	(1|Farm:Flock)	
11	Production space PC2 * Wild bird contact + Rotational (y/n)	(1|Farm:Flock)	
12	Soil organic matter * Wild bird contact + Rotational (y/n)	(1|Farm:Flock)	Excluded from final analyses due to missing data
13	Temperature * Wild bird contact + Rotational (y/n)	(1|Farm:Flock)	
14	Humidity * Wild bird contact + Rotational (y/n)	(1|Farm:Flock)	
15	Production type PC1 + Wild bird contact + Rotational (y/n)	(1|Farm:Flock)	
16	Production space PC2 + Wild bird contact + Rotational (y/n)	(1|Farm:Flock)	
17	Soil organic matter + Wild bird contact + Rotational (y/n)	(1|Farm:Flock)	Excluded from final analyses due to missing data
18	Temperature + Wild bird contact + Rotational (y/n)	(1|Farm:Flock)	

19	Humidity + Wild bird contact + Rotational (y/n)	(1|Farm:Flock)	
20	Production type PC1 * % agriculture (2100 m)	(1|Farm:Flock)	
21	Production space PC2 * % agriculture (2100 m)	(1|Farm:Flock)	
22	Soil organic matter * % agriculture (2100 m)	(1|Farm:Flock)	Excluded from final analyses due to missing data
23	Temperature * % agriculture (2100 m)	(1|Farm:Flock)	
24	Humidity * % agriculture (2100 m)	(1|Farm:Flock)	
25	Production type PC1 + % agriculture (2100 m)	(1|Farm:Flock)	
26	Production space PC2 + % agriculture (2100 m)	(1|Farm:Flock)	
27	Soil organic matter + % agriculture (2100 m)	(1|Farm:Flock)	Excluded from final analyses due to missing data
28	Temperature + % agriculture (2100 m)	(1|Farm:Flock)	
29	Humidity + % agriculture (2100 m)	(1|Farm:Flock)	
30	Production type PC1 * Wind speed	(1|Farm:Flock)	
31	Production space PC2 * Wind speed	(1|Farm:Flock)	
32	Soil organic matter * Wind speed	(1|Farm:Flock)	Excluded from final analyses due to missing data
33	Temperature * Wind speed	(1|Farm:Flock)	
34	Humidity * Wind speed	(1|Farm:Flock)	
35	Production type PC1 + Wind speed	(1|Farm:Flock)	
36	Production space PC2 + Wind speed	(1|Farm:Flock)	

37	Soil organic matter + Wind speed	(1|Farm:Flock)	Excluded from final analyses due to missing data
38	Temperature + Wind speed	(1|Farm:Flock)	
39	Humidity + Wind speed	(1|Farm:Flock)	
40	% agriculture (2100 m) * Wild bird contact + Rotational (y/n)	(1|Farm:Flock)	
41	% agriculture (2100 m) + Wild bird contact + Rotational (y/n)	(1|Farm:Flock)	
42	% agriculture (2100 m) * wind speed	(1|Farm:Flock)	
43	% agriculture (2100 m) + wind speed	(1|Farm:Flock)	

Table S2. Model selection results for factors that predict *Campylobacter* spp. prevalence in poultry feces using the subset of observations with soil data. Only models having > 5% of model weights are shown. Numbers in columns "Production type PC1" through "Production type PC1 * wind speed" indicate estimates ± standard error (SE). Flock nested within farm is included as a random effect. The next-best model not shown had $\Delta AIC_c = 4.6$ and weight = 0.049. Wind speed is the average wind speed over the 7 days preceding sampling. See Table S1 for the full set of models considered.

**Model**	**Production type PC1**	**Wind speed**	**Production type PC1 * Wind speed**	**% agriculture**	**ΔAIC_c***	**df**	**Weight**
Production type PC1 * Wind speed	0.22 ± 0.13 (SE) (*P* = 0.098)	**0.82 ± 0.20 (SE) (*P* < 0.0001)**	**0.63 ± 0.27 (SE) (*P* = 0.021)**		0	6	0.50
Wind speed + % agriculture		**0.52 ± 0.17 (SE) (*P* = 0.0028)**		**0.37 ± 0.17 (SE) (*P* = 0.037)**	3.0	5	0.11
Production type PC1 + Wind speed	0.22 ± 0.11 (SE) (*P* = 0.057)	**0.62 ± 0.16 (SE) (*P* = 0.00014)**			4.3	5	0.059

* Akaike Information Criterion with a correction for small sample sizes

Table S3. Model selection results for factors that predict *Campylobacter* spp. prevalence in poultry feces using all farms and not including soil parameters. Only models having > 5% of model weights are shown. Numbers in columns "Production type PC1" through "rotational (yes)" indicate estimates ± standard error (SE). Flock nested within farm is included as a random effect. The next-best model not shown had $\Delta AIC_c = 3.4$ and weight = 0.044. Wind speed = the average wind speed over the 7 days preceding sampling; % agriculture = amount of grazing and cropland in a 2100 m radius; rotational = flocks are rotated onto new pasture at 1+ times per year (used to account for wild bird contact on pasture not within enclosure area at the time of the survey). See Table S1 for the full set of models considered.

**Model**	**Production type PC1**	**Wind speed**	**Production type PC1 * Wind speed**	**% agriculture**	**Wild bird contact**	**Wild bird contact * % agriculture**	**Rotational (yes)**	**ΔAIC_c***	**df**	**Weight**
Production type PC1 + Wind speed	**0.29 ± 0.11 (SE) (*P* = 0.011)**	**0.52 ± 0.16 (SE) (*P* = 0.00087)**						0	5	0.24
Production type PC1 * Wind speed	**0.30 ± 0.13 (SE) (*P* = 0.020)**	**0.57 ± 0.17 (SE) (*P* = 0.00087)**	0.26 ± 0.21 (SE) (*P* = 0.22)					0.7	6	0.17
Production type PC1 + % agriculture	**0.36 ± 0.14 (SE) (*P* = 0.011)**			**0.53 ± 0.19 (SE) (*P* = 0.0066)**				1.0	5	0.14
Wind speed + % agriculture		**0.43 ± 0.16 (SE) (*P* = 0.0086)**		**0.33 ± 0.16 (SE) (*P* = 0.047)**				2.3	5	0.075
Wild bird contact * % agriculture + Rotational (yes)				**0.53 ± 0.27 (SE) (*P* = 0.045)**	0.38 ± 0.20 (SE) (*P* = 0.053)	-1.00 ± 0.56 (SE) (*P* = 0.073)	0.85 ± 0.57 (SE) (*P* = 0.13)	2.7	7	0.060

* Akaike Information Criterion with a correction for small sample sizes
